# Towards multiplexed immunofluorescence of 3D tissues

**DOI:** 10.1186/s13041-023-01027-9

**Published:** 2023-05-02

**Authors:** Wonjin Cho, Sehun Kim, Young-Gyun Park

**Affiliations:** Department of Bio and Brain Engineering, Korea Advanced Institute of Science and Technology, Daejeon, Republic of Korea

**Keywords:** Immunofluorescence, Multiplexed immunofluorescence, 3D immunostaining

## Abstract

Profiling molecular expression in situ allows the integration of biomolecular and cellular features, enabling an in-depth understanding of biological systems. Multiplexed immunofluorescence methods can visualize tens to hundreds of proteins from individual tissue samples, but their application is usually limited to thin tissue sections. Multiplexed immunofluorescence of thick tissues or intact organs will enable high-throughput profiling of cellular protein expression within 3D tissue architectures (e.g., blood vessels, neural projections, tumors), opening a new dimension in diverse biological research and medical applications. We will review current multiplexed immunofluorescence methods and discuss possible approaches and challenges to achieve 3D multiplexed immunofluorescence.

## Main text

Immunofluorescence (IF) can visualize proteins in tissues using antibodies and fluorophores [1]. The number of proteins IF can visualize from individual tissue is limited to 4–5 due to the spectral overlapping of fluorophores [1, 2].

Multiplexed IF methods can visualize tens to hundreds of proteins from each tissue [1, 3–8], enabling in-depth analysis of tissues in diverse fundamental and clinical research. However, multiplexed IF methods are currently limited to thin tissue section [1, 2, 7]. 3D multiplexed IF, multiplexed IF of millimeter-thick tissues or intact organs, can be achieved by developing a multiplexed IF method applicable to 3D tissues and by visualizing immunolabeled 3D tissues using tissue clearing and 3D microscopy techniques.

Here we will review current multiplexed IF methods and 3D immunostaining methods. Then we will discuss possible approaches and challenges to realize 3D multiplexed IF.

### Current multiplexed IF methods

**Fig. 1:**
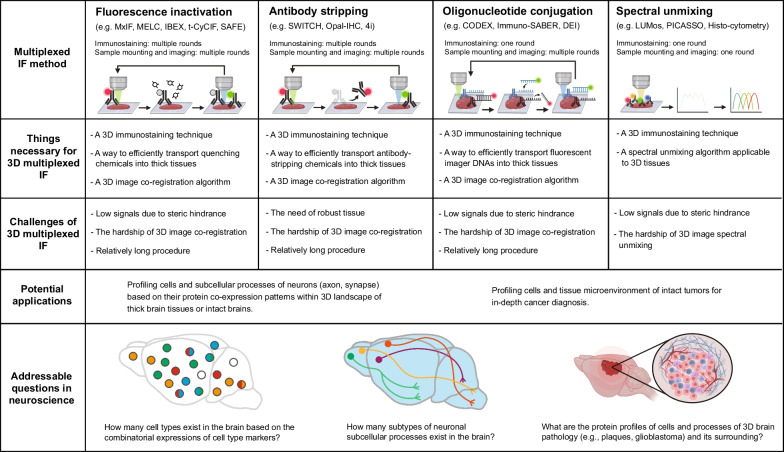
Potential approaches and challenges to achieve 3D multiplexed IF with its impacts

Current methods can be divided into four categories: fluorescence inactivation, antibody stripping, oligonucleotide conjugation, and spectral unmixing methods (Fig. 1). Among those, fluorophore inactivation and antibody stripping methods can be collectively termed “Cyclic IF” as those involve multiple cycles of immunostaining, imaging, removal of fluorescence signals, and re-immunostaining with another set of antibodies [1].

A fluorophore inactivation method removes fluorescence signals by quenching fluorophores via photobleaching, fluorophore oxidation, or fluorophore unconjugation [1, 3]. This method allows removing fluorescence signals with mild processes compared to antibody stripping methods [1, 3]. However, this method can suffer from low signal quality caused by steric hindrance among antibodies because residual bound antibodies from the previous rounds can interfere with antibody binding of the next rounds [9].

An antibody stripping method removes fluorescence signals by detaching antibodies from tissues [4, 5]. Unlike all the other methods, this method is free from the steric hindrance problem. However, the antibody stripping process can deform tissue and damage tissue antigenicity [5], which can be ameliorated by using tissue protection techniques [5, 10].

An oligonucleotide conjugation method utilizes DNA-barcoded antibodies [1, 6]. Tissue is simultaneously immunolabeled with tens of different antibodies conjugated with unique DNA oligomers of which subset can be visualized by using fluorescent imager DNAs. This method needs just a single round of immunostaining, although requires multiple rounds of labeling with different sets of imager DNAs [6]. Moreover, steric hindrance can happen among tens of different antibodies competing for binding during immunostaining [9].

A spectral unmixing method involves imaging tens of antibodies with overlapping fluorescence spectra followed by unmixing the spectra using a computational algorithm. This method is simple because it requires only one round of immunostaining and sample mounting for imaging [7, 8], which exempts this method from the need for image co-registration (registering images generated from iterative rounds of sample mounting and imaging of the same tissue) that is necessary for all the other methods. However, a spectral unmixing method highly depends on the accuracy of the spectral unmixing algorithm that is sensitive to tissue properties, imaging conditions, and microscope setup. This method also suffers from the steric hindrance happening during immunostaining with tens of antibodies [9].

### Current 3D immunostaining methods

3D multiplexed IF requires uniformly immunostain 3D tissues. Current 3D immunostaining methods can be categorized based on the strategies facilitating antibody transport into tissues: increasing antibody diffusion rate, decreasing antibody reaction rate, or the combination of both.

Increasing antibody diffusion rate has been achieved by increasing tissue pore sizes using chemicals [11], or by utilizing forces that facilitate diffusion. For example, ACT-PRESTO utilizes centrifugal force [12], and stochastic electrotransport utilizes an electric field [13].

Another approach for 3D immunostaining involves decreasing antibody reaction rate, which in turn increases the distances that antibodies travel into a tissue before binding to target proteins, facilitating antibody transport into tissue [2]. For example, CUBIC-HistoVIsion utilizes quadrol and urea to attenuate antibody binding [11].

There is a method combining both approaches. eFLASH increases antibody diffusion rate by using stochastic electrophoresis while reducing antibody reaction rate using pH and a detergent [14]. Then the gradual change of pH and the concentration of the detergent allows distributed antibodies to bind to nearby target proteins, achieving uniform immunostaining of 3D tissues within 1–2 days [14].

### Potential approaches and challenges to achieve 3D multiplexed IF

3D multiplexed IF requires 3D immunostaining combined with a strategy for multiplexing as well as techniques for visualization of labeled 3D tissues. Multiplexing strategies of the fluorescence inactivation and antibody stripping methods can be scalable to 3D tissues by engendering ways to transfer quenching and antibody-stripping chemicals into 3D tissues, respectively. The multiplexing strategy of the oligonucleotide conjugation method is applicable to 3D tissues by devising a way to efficiently transport imager DNAs into thick tissues. A spectral unmixing algorithm that is applicable to 3D tissue images can enable spectral unmixing-based 3D multiplexed IF. However, several challenges exist on achieving 3D multiplexed IF.

*Steric hindrance problem* the steric hindrance problem can lower signal quality [9], although high signal quality is crucial for 3D IF because high levels of light scattering and absorption of thick tissue imaging will reduce detectability of fluorescence signals. This problem can be overcome by adapting signal amplification methods, utilizing tissue clearing method that preserves proteins inside tissues, or using microscopy that minimizes photobleaching during imaging such as two-photon or light-sheet microscopy. The antibody stripping-based 3D multiplexed IF will be free from this problem [7].

*The hardship of 3D image co-registration* Except for the spectral unmixing method, the other three multiplexing methods need an image co-registration algorithm to integrate results from multiple images acquired from a single tissue, indicating the need of 3D image co-registration for 3D multiplexed IF. The level of the co-registration needs to be cellular in order to access protein profiles from individual cells of 3D tissues. The 3D image co-registration with cellular resolution requires diverse technological invention including a high-fidelity 3D image co-registration algorithm, a tissue clearing technique that protects tissue architecture while multiplexing, and tissue clearing and 3D imaging techniques that provide high-quality 3D images with minimal spherical aberration.

*Long procedure* the duration of 3D multiplexed IF is crucial for the throughput and depth of multiplexing. The duration can be shorter by utilizing fast tissue clearing, 3D microscopy (e.g., light-sheet microscope), and 3D immunostaining methods. 3D multiplexed IF based on oligonucleotide conjugation or spectral unmixing could be shorter than other methods since it needs only one round of immunostaining.

### Outlook and prospects

Many challenges exist on realizing 3D multiplexed IF in addition to the general hardship of tissue clearing, 3D tissue imaging, 3D immunostaining, and 3D image analysis. However, once realized, 3D multiplexed IF will answer fundamental questions of neuroscience and will enable novel translational applications (Fig. 1).

## Data Availability

Not applicable. No data was generated during the current study.

## Abbreviations

3D: Three-dimensional
DNA: Deoxyribonucleic acid
